# Translating microbial knowledge into clinical biofilm therapies

**DOI:** 10.1515/med-2026-1544

**Published:** 2026-09-29

**Authors:** Abdulaziz Al Mouslem, Ghallab Alotaibi, Abdullah Alkhammash, Shinu Pottathil

**Affiliations:** Department of Pharmaceutical Sciences, College of Clinical Pharmacy, King Faisal University, Al-Ahsa, Saudi Arabia; Department of Pharmacology, College of Pharmacy, Al-Dawadmi Campus, Shaqra University, Shaqra, Saudi Arabia; Department of Biomedical Sciences, College of Clinical Pharmacy, King Faisal University, Al-Ahsa, Saudi Arabia

**Keywords:** biofilms, extracellular polymeric substances, antibiofilm strategies, nanotechnology, microfluidics, antimicrobial resistance

## Abstract

Biofilms are highly organized microbial communities that adhere to living or nonliving surfaces and are enclosed in a self-produced extracellular matrix. Their tolerance against antimicrobial agents and host defenses makes them a major challenge across clinical, industrial, and environmental fields. This review offers an in-depth analysis of biofilm formation, structure, and dispersal mechanisms, highlighting the genetic and molecular regulators that underlie their development. This review also provides extensive details of advanced and investigative strategies for biofilm eradication, including antibody-based approaches, photodynamic therapy, nanotechnology-driven solutions, surface modification of biomaterials, biological and phage-based approaches, the integration of smart and responsive systems, and host immune modulation methods. By integrating recent advances in imaging, experimental modeling, therapeutic innovations, artificial intelligence, and machine learning models, this work highlights not only the complexity of biofilm biology but also the research and development prospects for effective intervention tools. Finally, this review discusses major translational challenges associated with moving antibiofilm strategies from laboratory research to clinical and device-related applications. This review also proposes practical guidelines to overcome these challenges. Overall, the review highlights the requirements for interdisciplinary collaboration across microbiology, materials science, clinical medicine, and regulatory frameworks. Unlike traditional descriptive reviews, this review adopts a decision-oriented framework, providing clear research guidance for researchers.

## Introduction

Biofilms are structured microbial communities that adhere irreversibly to a surface and are encased in a self-produced extracellular polysaccharide matrix [1]. Biofilm-producing bacteria differ from free-floating planktonic bacteria in the production of extracellular polymeric substances (EPS), reduced growth rates, and altered gene regulation patterns [2]. Mature biofilms provide a suitable environment for intercellular communication and horizontal gene transfer [3].

Currently, biofilms pose a key challenge in antimicrobial therapy. There is a fundamental difference between phenotypic antibiotic tolerance, which is exhibited by bacteria within biofilms, and genetic antibiotic resistance, acquired by planktonic bacteria through gene mutations or horizontal gene transfer. The causes of biofilm-mediated phenotypic tolerance include reduced metabolic activity, limited penetration of antibacterial agents, altered microenvironmental conditions, the presence of persister bacteria within the matrix, and the protective effect of the EPS matrix. Nutrient limitation also plays a vital role in enhancing antimicrobial tolerance, as starvation conditions stimulate increased polysaccharide production [4]. The heterogeneous microenvironment formed by oxygen gradients leaves deep-layer bacteria in a long-term hypoxic state, with a growth rate far lower than that of surface-layer bacteria, making them difficult to eliminate [5]. This type of tolerance triggers various device-related infections [1], and also induces infections caused by *Streptococcus mutans* (*S. mutans*) and *Staphylococcus aureus* (*S. aureus*) [6], posing enormous challenges to clinical practice [6]. While much progress has been made in studies of mechanisms of biofilm formation, the translation of these basic research findings into effective clinical therapies remains very limited. Anti-biofilm strategies that deliver prominent efficacy in laboratory settings fail to replicate the same effect in patients. The core barriers to this translation include complex host microenvironments, polymicrobial interactions, immune regulation, and heterogeneity across infection sites [7]. Clinically, biofilm-associated infections remain a major challenge in orthopedic implant infections, catheter-associated infections, chronic diabetic wounds, cystic fibrosis-associated respiratory infections, and prosthetic joint infections (PJIs), where persistent biofilms contribute to recurrent infection, delayed healing, prolonged hospitalization, and increased antimicrobial use [8]. Furthermore, only a limited number of antibiofilm interventions have progressed into randomized clinical studies, and standardized clinical treatment protocols remain largely unavailable [8]. These translational limitations emphasize the need for clinically relevant multispecies biofilm models, stronger integration of clinical trial evidence, and multidisciplinary collaboration between microbiologists, clinicians, biomaterial scientists, and regulatory agencies to improve the successful implementation of antibiofilm therapies in healthcare settings [9].

The current review is organized around a translational framework that connects basic biofilm biology with clinical therapeutic development. This review first summarizes the underlying mechanisms of biofilms, followed by experimental and analytical models used to investigate biofilm-associated infections; a critical evaluation of conventional and emerging antibiofilm strategies; and, finally, key translational challenges and future directions of antibiofilm development.

### Literature search strategy

This article is a narrative review with the main objective of providing a translational research perspective on biofilm biology and emerging anti-biofilm therapies. We selected four major academic databases such as PubMed, Scopus, Web of Science, and Google Scholar, and searched using interrelated keyword combinations including “biofilm,” “biofilm-associated infections,” “antibiofilm therapy,” “antimicrobial tolerance,” “biofilm resistance,” “nanotechnology,” “photodynamic therapy,” “bacteriophage therapy,” “quorum sensing,” “medical device infections,” and “clinical translation”. We prioritized the inclusion of English-published peer-reviewed research articles, recent reviews, clinical studies, and translational research, and all selected literature was required to align with its relevance to biofilm mechanisms, therapeutic strategies, experimental models, translational challenges, and clinical applicability.

## Biofilm formation and structure

Biofilm formation is a complex, multifaceted process that involves genetic regulation, signaling networks, and environmental cues. Various studies have noted the roles of genes encoding efflux pumps, pili, and quorum-sensing systems in biofilm development [4], 10]. The biofilm life cycle is typically described in four stages, as shown in Figure 1: initial adhesion, microcolony formation, maturation, and eventual dispersal [11].

**Figure 1:**
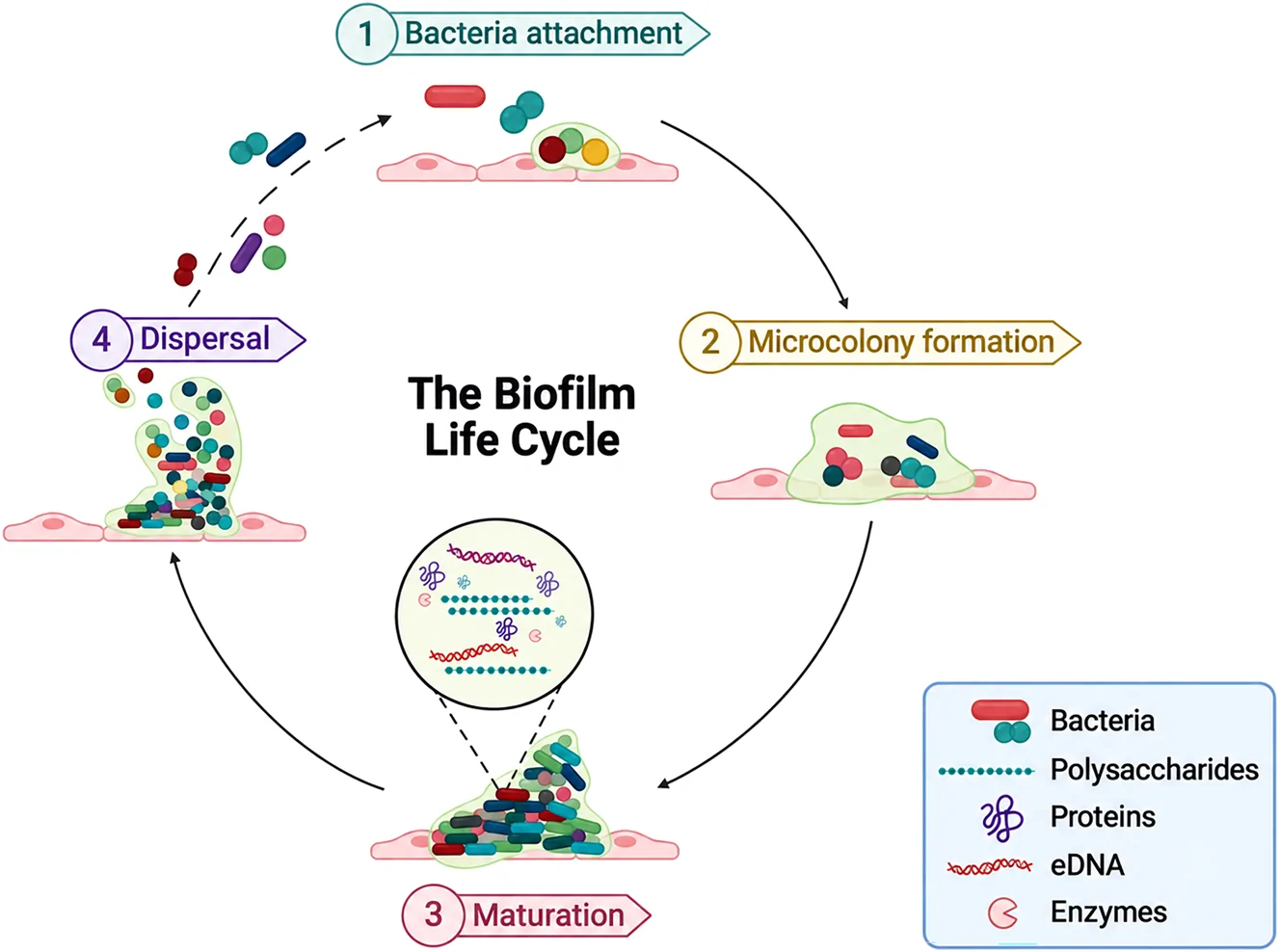
A schematic diagram illustrating the life cycle of biofilms, which covers the four consecutive stages of initial bacterial attachment, microcolony formation, maturation, and dispersion. This figure is reproduced from Montanari et al. [12] under the terms of the Creative Commons Attribution 4.0 International License (CC BY 4.0).

### Adhesion

The earliest stage of biofilm formation begins with the attachment of microorganisms to surfaces. This process is influenced by environmental and physical forces, including hydrodynamics, gravitational force, and Brownian motion, which lead to unpredictable bacterial colonization. To mediate adhesion, bacteria must rely on extracellular appendages and secreted adhesins to overcome interfacial repulsive forces; the surface proteins involved in this process include Protein A, SasG, the biofilm-associated protein (Bap), and outer membrane protein A (OmpA) [1]. It was previously believed that chemotaxis is non-essential for *Escherichia coli* (*E. coli*). However, defects in chemotaxis-related proteins of uropathogenic *E. coli* (UPEC) disrupt its biofilm development [13].

### Maturation

After microorganisms establish surface contact, they initiate genetic reprogramming to shift to a sessile survival mode and upregulate matrix-related proteins and extracellular components to enhance community stability. The extracellular matrix of *Pseudomonas aeruginosa (P. aeruginosa)* can adapt to its surrounding environment; small-colony variants of this bacterium isolated from cystic fibrosis patients exhibit upregulated *pel* gene expression, which enhances the strain’s ability to persist in the host’s airway [14]. Apart from extracellular matrix, extracellular deoxyribonucleic acids (eDNA) have emerged as an essential stabilizing factor in biofilm architecture that supports cell-to-cell interactions [15]. In *Enterococcus faecalis (E. faecalis)*, eDNA maintains biofilm structural integrity, while the zinc metalloprotease GelE and serine protease SprE regulate autolysis and DNA release, thereby influencing biofilm development [16], 17].

### Dispersal

Mature biofilms are dynamic ecosystems that support cell-to-cell interactions, nutrient exchange, and responses to environmental changes. Biofilm dispersal is essential for their expansion, and these dispersal mechanisms fall into two categories: passive dispersion, which is triggered by shear stress, and active dispersion, which is triggered by environmental signals such as nutrient and oxygen gradients, or toxic metabolite accumulation [1], 18].

UPEC and *P. aeruginosa* each have distinct biofilm dispersion trigger signals such as a surge in extracellular iron and changes in nitrogen and carbon supply, respectively. [19]. The BdcA protein reduces EPS synthesis and increases bacterial motility by regulating intracellular cyclic di-guanosine monophosphate (c-di-GMP) levels in both bacteria [20], 21]. Surfactant production also contributes to dispersal; in *P. aeruginosa*, rhamnolipids aid channel formation within mature biofilm at low concentrations but drive dispersion when overproduced [22]. Furthermore, programmed cell death within the biofilm generates hollow regions, releasing viable cells into the environment where they can reinitiate planktonic growth under favorable conditions [23].

## Experimental and analytical approaches for biofilm research

To capture the structural and functional complexity of biofilms, biofilm research must meet two core requirements: reliable and reproducible experimental models, and high-end analytical tools. Over the years, several groups have reported various sets of *in vitro* models ranging from simple agar colonies to complex microfluidic platforms, each designed to answer specific biological or clinical problems [23], 24]. When combined with staining, imaging, and spectrophotometry, these models detect four core indicators, including microbial viability, extracellular matrix composition, metabolic activity, and biofilm resilience, which supports the deepening of basic research and the translation of research outcomes.

### Traditional culture-based models

The agar plate-based bacterial colony model is easy to operate and has extremely high research value. It replicates the two core characteristics of biofilms: high cell density and nutrient gradient [25] and serves as a phenotypic marker for genetic regulatory networks in *Bacillus subtilis* (*B. subtilis*) research [25], 26]. This model also simulates biofilms in soft tissue infections and cystic fibrosis, since this model acquires nutrients via direct uptake from the substrate [27].

The microtiter plate assay is a fundamental technique in biofilm research. After biofilm-forming bacteria adhere to the walls of polystyrene wells, the total biomass is measured using crystal violet staining [28]. This method is used for high-throughput antimicrobial drug screening, testing biofilm formation across strains, and research on hydroxyapatite substrates for oral biofilms [29]. While it fails to simulate *in vivo* fluid dynamics and nutrient gradients, it carries the combined advantages of high reproducibility and low cost [28].

### Flow-based reactor systems

Advanced biofilm models that integrate continuous flow and hydrodynamic shear and are capable of simulating real environmental conditions. The CDC biofilm reactor, a standardized platform in this field, offers controllable nutrient flow and removable test coupons. It produces repeatable samples that meet clinical and industrial requirements and is used to evaluate antimicrobial agents, disinfectants, and biomaterial surfaces [30]. This reactor is also used to conduct research on urinary tract device encrustation and to study *Listeria monocytogenes* in the food industry. When paired with Confocal laser scanning microscopy (CLSM), the CDC biofilm reactor reveals the surface properties and incubation conditions affecting bacterial adhesion [31], 32].

The narrow laminar-flow environment of Capillary biofilm reactors (CBRs) makes these systems ideal tools for researching mixed-species communities. CBRs are compatible with species-specific fluorescent labeling and CLSM [33], and have been applied in studies of cyanobacterial biofilms. However, their long-term use is limited due to low stability and insufficient production capacity [34].

Rotating disk reactors (RDRs) simulate controlled shear environments and are suitable for studying adhesion and detachment behaviors of biofilms on material surfaces. For example, rheological studies of *P. aeruginosa* biofilms revealed strong viscoelastic properties under mechanical stress [35]. Similarly, Drip-flow reactors (DFRs) maintain heterogeneous biofilms at the gas-liquid interface and are well-adapted for translational antimicrobial testing suited to wound and oral cavity infections [36], 37].

### Microfluidic and next-generation systems

Microfluidic devices are a frontier technology in current biofilm modeling, capable of precisely regulating microenvironmental factors such as fluid dynamics and nutrient gradients [38]. These platforms are integrated with biosensors and CLSM, enabling real-time monitoring of biofilm adhesion, heterogeneity, and dispersal [39]. Applications include mimicking vascular systems for bloodstream infections and reproducing mucosal or wound environments. While fabrication complexity limits widespread use, microfluidics is poised to transform both basic biofilm research and high-throughput drug discovery [40]. All types of experimental biofilm models are detailed in Table 1.

**Table 1:** Comparative summary of experimental biofilm models.

Model type	Model	Cost	Throughput	Physiological relevance	Species complexity	Imaging compatibility	Key limitations	Best application
Static [25], [26], [27], [28], [29]	Agar plate	Low	High	Low	Low	Low (macroscopic only)	Poor control over nutrient gradients	Initial biofilm formation studies
	Microtiter plate	Low	High	Moderate	Low to moderate	Moderate (e.g., staining)	Oxygen/nutrient depletion over time	Adhesion studies, high-throughput screening
	Hydroxyapatite discs	Moderate	Moderate	High (oral/bone)	Low	Moderate	Poor model for soft tissue or catheter-associated infections	Oral and dental biofilm studies
Flow-based [30], [31], [32], [33], [34], [35], [36], [37]	CDC biofilm reactor	High	Moderate	High	High	High (CLSM compatible)	Expensive and complex setup	Device-associated biofilms
	Rotating disk reactor (RDR)	Moderate	Moderate	High	Moderate	Moderate to high	Uneven shear conditions, therefore, may not reflect all clinical environments	Device-associated biofilms and coating evaluation
	Drip flow reactor (DFR)	Moderate	Moderate	High (air-liquid)	Moderate	High	Restricted to surface-associated biofilms, so it may not be suitable for studying heart valves or high-pressure catheters	Matrix penetration, maturation, dispersal
	Capillary biofilm reactor	High	Low	Moderate	High	High (CLSM, FISH)	Technically complex and costly	Flow-based biofilm growth, penetration, and dispersal studies
Microfluidic [38], [39], [40], [41], [42], [43], [44], [45]	Custom microfluidic devices	High	Low to moderate	High	High	High (real-time CLSM)	Bubbles can easily destroy the biofilm in small channels	Host–pathogen interactions, real-time monitoring
	Organ-on-a-chip systems	High	Low	High	High	High (real-time imaging, CLSM, live-cell analysis)	High cost, technical complexity, limited standardization, and low scalability for high-throughput screening	Translational and host-integrated studies

Low, moderate, and high indicate relative qualitative comparisons among biofilm models based on operational cost, throughput, physiological relevance, and imaging compatibility. Low=basic setup, limited throughput, minimal *in vivo* simulation, and basic microscopy; Moderate=specialized equipment, intermediate throughput, partial physiological simulation, and fluorescence-compatible imaging; High=advanced instrumentation, high-throughput capability, close *in vivo* resemblance, and CLSM/live high-resolution imaging compatibility.

New host-integrated biofilm models developed based on organ-on-a-chip technology can accurately reproduce physiological microenvironments, hydrodynamic conditions, and host-pathogen interactions [41]. These models have been used to successfully construct complex human physiological barriers, including the blood-brain barrier and airway microenvironments. They support research on host-microbe interactions under dynamic conditions, deliver better translational relevance than traditional models, and can improve the ability to predict *in vivo* therapeutic effects. [42], 43]. However, these models are constrained by four core bottlenecks: technical complexity, standardization, scalability, and accessibility, so they cannot replace traditional models for the time being. The traditional biofilm models (such as microtiter plates and CDC reactors) continue to serve as valuable platforms for mechanistic studies and therapeutic screening; they often lack important features of clinical infections, such as polymicrobial complexity, host immune interactions, tissue architecture, nutrient gradients, shear stress, and microenvironmental heterogeneity observed *in vivo* [44]. Consequently, discrepancies frequently arise between laboratory efficacy and clinical outcomes, limiting the translational predictability of experimental findings [45]. Therefore, improving the clinical relevance of experimental biofilm models is a core prerequisite to accelerate the translation of anti-biofilm therapies.

### Staining and imaging methods

The visualization and quantification of biofilms rely heavily on advanced staining and microscopy. Fluorescent assays such as BacLight™ Live/Dead (SYTO 9 and propidium iodide) enable distinction between viable and non-viable bacterial cells [46], while acridine orange distinguishes DNA- and RNA-rich cells to identify metabolically active microbial cells [47]. The methylene blue assay is useful for general biofilm visualization and biomass assessment, but lacks intrinsic species specificity unless combined with complementary molecular methods [48]. At the same time, Fluorescein diacetate (FDA) provides an effective means of assessing cell viability, producing bright yellow fluorescence upon enzymatic cleavage by viable bacteria, thereby marking only metabolically active populations [49]. For extracellular matrix analysis, stains like calcofluor white (binds to β-polysaccharides) and concanavalin A conjugates (bind to α-mannopyranosyl and α-glucopyranosyl residues) identify carbohydrate-rich domains within the matrix, while fluorescein-labeled lectins distinguish between different sugar residues, achieving species-level resolution of EPS heterogeneity [50], 51].

CLSM remains one of the most widely utilized techniques for three-dimensional biofilm visualization and quantitative structural analysis, although complementary methods are often required for species identification and functional characterization [52]. The techniques, such as Fluorescent *in situ* hybridization (FISH) enable microbial species identification [53], while time-lapse CLSM provides dynamic monitoring of biofilm development. Complementary methods include Scanning electron microscopy (SEM), Transmission electron microscopy (TEM), scanning probe microscopy, X-ray imaging, and mass spectrometry-based imaging for nanoscale and compositional insights, revealing the distribution of biomolecules and metabolites across biofilm layers [54]. Table 2 shows a comparative overview of biofilm imaging and analytical methods: readouts, capabilities, limitations, and recommended applications.

**Table 2:** Comparative overview of biofilm imaging and analytical methods: readouts, capabilities, limitations, and recommended applications.

Method	Readout type	Spatial resolution	Live/dead compatibility	Matrix information	Species specificity	Common limitation	Best use scenario
Crystal violet [55], 56]	Total biomass	Low	No	Limited	No	Do not distinguish live/dead cells	Biomass quantification
Methylene blue [48], 56]	Biomass/cell staining	Low	No	Limited	No	Nonspecific staining; poor species discrimination	Preliminary biofilm assessment
Acridine orange [47]	Total cell visualization (DNA/RNA staining)	Moderate	Limited	No	No	Do not reliably distinguish viable from non-viable cells; nonspecific nucleic acid staining	General biofilm visualization and cell distribution studies
Fluorescein diacetate (FDA) [49], [50], [51]	Metabolic activity/viability	Low–moderate	Yes (indirect)	No	No	Esterase activity varies among species; it may underestimate dormant cells	Assessment of overall metabolic activity
Calcofluor white [50], 51]	Polysaccharide and EPS visualization	Moderate	No	Yes	No	Binds specific polysaccharides only; may not detect all matrix components	EPS and biofilm matrix characterization
SYTO/PI (live/dead assay) [46]	Cell viability and membrane integrity	Moderate–high	Yes	Limited	No	Dye penetration bias: extracellular DNA may affect interpretation	Live/dead cell distributionwithin biofilms
Mass spectrometry imaging (MSI) [59]	Spatial distribution of metabolites, drugs, and biomolecules	High	No	Indirect	Limited to moderate	Expensive instrumentation; complex data analysis; limited routine use	Mapping antimicrobial penetration and metabolic heterogeneity
CLSM [52]	Three-dimensional biofilm architecture	High	Yes	Moderate	High when paired with FISH; otherwise limited	Photobleaching; limited imaging depth in thick biofilms	Structural and viability analysis
FISH [53]	Species identification and spatial organization	High	Limited	No	High	Probe penetration limitations; requires prior sequence knowledge	Polymicrobial biofilm analysis
SEM/TEM [54]	Surface topography/internal cross-sections	High	No	Limited	No	Dehydration artifacts and sample preparation effects	Surface ultrastructure characterization

Low, moderate, and high indicate relative performance among methods. The readout type refers to the primary biological information of the biofilm. Spatial resolution refers to structural details within biofilms; live/dead compatibility indicates the capacity to differentiate viable and non-viable cells; matrix information reflects characterization of extracellular polymeric substance; and species specificity denotes the ability to distinguish individual microbes within polymicrobial biofilm communities.

Although various imaging and staining technologies provide effective support for research on the structure, activity, and composition of biofilms, they all carry inherent limitations that interfere with data interpretation. Conventional crystal violet and methylene blue staining fail to yield detailed information on spatial organization, metabolic activity, and species composition [55], 56]; fluorescent activity detection methods produce inaccurate estimates of live and dead bacteria due to incomplete dye penetration [57]. CLSM fails to distinguish between test samples’ physiological activity and structural characteristics, as it is limited by insufficient imaging depth, restrictions on fluorophore selection, and photobleaching issues [58]. Consequently, the selection of analytical methods must be guided by the specific research objective, and only by integrating complementary staining and imaging approaches can a comprehensive characterization of clinically relevant biofilms be achieved.

### Cytotoxicity and *in silico* approaches

Cytotoxicity assessment is a key step to confirm the safety of antibiofilm agents before clinical translation. A combination of staining techniques (Trypan blue, crystal violet, DAPI, PI) and metabolic assays (MTT, XTT, resazurin) is commonly used to evaluate dose-dependent effects on mammalian cells [60]. The American Society for Testing and Materials (ASTM) guidelines provide standardized protocols to enhance reproducibility. Antibiofilm agents, such as octenidine hydrochloride, have demonstrated no evidence of mutagenic, carcinogenic, or genotoxic effects and no harmful impact on the gastrointestinal tract or mucosal membranes when tested *in vitro*, either alone or in oral formulations. However, the toxicity of Antimicrobial peptides (AMPs) and some synthetic antibiofilm agents varies with concentration and target cell types [61]. Computer simulation models help in the discovery of antibiofilm agents through molecular docking, quantitative structure-activity relationship (QSAR), and machine learning-based models [62]. Molecular dynamics (MD) simulations provide atomic-level insights, while network modeling captures the ecological complexity of biofilms [63], 64]. These tools bridge computational prediction and experimental validation and are suitable for both synthetic and natural products [65].

### How model choice shapes translational interpretation

The selection of experimental biofilm models directly affects mechanistic insights as well as the evaluation outcomes of antibiofilm strategies. Therefore, models must be precisely matched to specific research goals. There are three types of antibiofilm models, and each comprises its own advantages and disadvantages depending on its clinical relevance. First, static models, microtiter plate and agar-based assays, are easy to operate and suitable for research, such as investigating initial adhesion mechanisms and primary screening of compounds, but fail to simulate the core characteristics of clinical biofilms, such as hydrodynamic conditions, nutrients, and oxygen gradients. Therefore, these models may overestimate the penetration rate of antimicrobial agents and therapeutic efficacy [44]. Second, flow-based models, CDC biofilm reactors, and capillary reactors have far greater clinical compatibility than static models, thereby providing a more clinically relevant platform to evaluate antimicrobial coatings and biofilm tolerance to therapeutic agents [66]. Third, the microfluidic and organ-on-chip platforms provide a higher degree of physiological relevance by enabling real-time monitoring of host–pathogen interactions, polymicrobial dynamics, and spatial heterogeneity within biofilm communities [67]. The microfluidic systems are better suited for investigating matrix penetration, biofilm maturation, dispersal dynamics, and therapeutic delivery under realistic environmental conditions. Similarly, host-integrated models more accurately mimic chronic wounds, oral biofilms, and respiratory tract infections than conventional mono-species cultures (Table 2) [44]. Consequently, translational interpretation of antibiofilm efficacy should always consider the limitations and ecological relevance of the underlying model system. Failure to do so may result in therapeutic strategies that perform well *in vitro* but fail to demonstrate comparable effectiveness in clinical settings [68].

## Biofilm-associated antimicrobial tolerance, resistance, and therapeutic strategies

Bacterial biofilms play a central role in chronic and persistent infections because they confer enhanced antimicrobial tolerance and immune evasion capabilities. Biofilm-associated tolerance is multifactorial and differs from classical genetic antibiotic resistance. It primarily results from the EPS barrier, nutrient and oxygen gradients, reduced metabolic activity, stress-response pathways, and persister cell formation [69]. A defining feature of biofilm is the EPS matrix, which slows antibiotic penetration and alters the duration and efficacy of antimicrobial exposure. While traditional mechanisms of antibiotic resistance, such as efflux pumps, enzymatic degradation, and mutational changes, are well established in planktonic cells, biofilm-forming bacteria exhibit additional protective strategies. Within mature biofilms, gradients of oxygen and nutrients create zones of low metabolic activity, where dormant persistent bacterial cells exist. These cells are naturally tolerant to antibiotics since most antibiotics specifically target actively dividing cells [70], 71]. Biofilms can also act as bacterial reservoirs, and the bacteria they release can cause osteomyelitis, infective endocarditis, and PJIs [72]. Furthermore, these dispersed bacteria often regain antimicrobial sensitivity, which confirms that this tolerance is a reversible phenotypic trait, rather than a permanent genetic mutation [73].

Accurate differentiation among antimicrobial resistance, tolerance, persistence, and EPS-mediated protection is essential to understand the mechanisms underlying biofilm-associated treatment failure. Antimicrobial resistance is a heritable genetic adaptation that enables microorganisms to grow at antimicrobial concentrations that normally inhibit susceptible strains; antimicrobial tolerance refers to the survival of a microbial population after short-term exposure to a drug, with no increase in its minimum inhibitory concentration (MIC); Persister cells are a dormant, highly tolerant subpopulation of bacterial cells, capable of recolonizing biofilms after antimicrobial treatment withdrawal [74]; viable but non-culturable (VBNC) cells retain metabolic activity but lose the ability to grow under standard culture conditions [75]; EPS matrix-mediated protection limits drug penetration, generates physicochemical gradients, and induces phenotypic tolerance [76]. Although all five mechanisms collectively lead to biofilm recalcitrance, their biological bases and therapeutic significance differ fundamentally.

Alterations to the internal microenvironment of biofilms exacerbate pathogens’ antimicrobial tolerance. Changes in the internal pH, oxygen content, and nutrient availability within biofilms impede the penetration of antimicrobial drugs, reduce the stability and activity of these agents, and this effect is particularly pronounced under acidic and hypoxic conditions [77], 78]. After pathogens bind to the protective EPS matrix of biofilms, they are extremely difficult to eradicate even with potent antimicrobial therapy, and cause persistent infections. Implantable medical devices represent a high-risk scenario for this type of infection. After *S. aureus* colonizes these devices, it triggers inflammation and local acidification that delay healing, leaving the removal of the implanted device as the only viable solution. Multidrug-resistant strains, including methicillin-resistant *S. aureus* (MRSA) and vancomycin-resistant *S. aureus* (VRSA), further exacerbate the clinical burden [79]. These strains, capable of forming biofilms, are the primary cause of various medical device-associated infections and are directly linked to higher treatment failure rates, prolonged hospital stays, and increased mortality. Biofilm-related antimicrobial tolerance is currently imposing a growing global burden [80], 81].

In general, antibiofilm strategies can be classified as traditional, advanced, and emerging. The traditional approaches to biofilm management include surgical debridement, antibiotic-coated implants, and chemical agents that disrupt biofilms. These measures delay colonization or reduce biomass, but are often insufficient for complete eradication. Antibacterial coatings lose their efficacy as their active ingredients leach out, while chemical approaches often cause damage to normal tissue. The only currently reliable solution, which is implant removal, increases patients’ physical, psychological, and economic burden as well [82]. Figure 2 illustrates the formation mechanisms of biofilm-associated antimicrobial tolerance and drug resistance.

**Figure 2:**
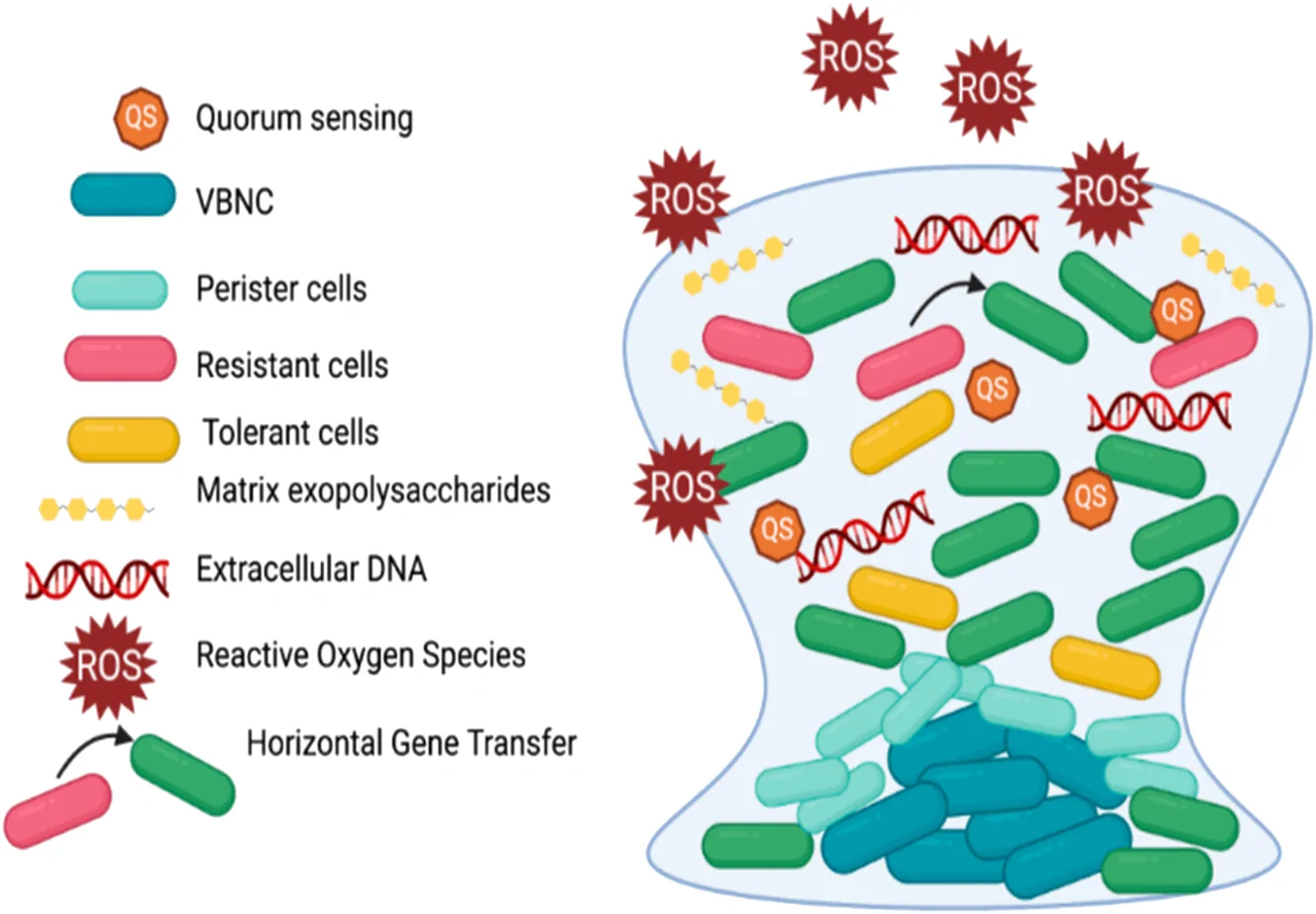
Mechanisms contributing to biofilm-associated antimicrobial tolerance and resistance. This figure is reproduced from Omwenga and Awuor [83] under the terms of the Creative Commons Attribution 4.0 International License (CC BY 4.0).

To overcome various limitations in current biofilm research, a stage-specific targeted intervention approach (targeting adhesion, maturation, and dispersal stages of biofilm formation) has been developed. During the adhesion phase, various lytic enzymes (such as DNases, proteases, and hydrolases) to interfere with initial attachment [84]. In the maturation phase, destabilization of EPS and disruption of biofilm structure using antibiofilm agents; once biofilms reach the dispersal phase, use of innovative interventions such as nanomaterials, multifunctional compounds, ultrasound, photothermal therapy, and immunotherapy to prevent secondary colonization may be helpful [85]. Advanced Strategies have expanded beyond conventional antibiotics to include combination therapies, which simultaneously target metabolically active and dormant subpopulations. Such combined antimicrobial therapy has better therapeutic effects than single-drug treatment [86]. Surface modifications with hydrophilic polymers like polyethylene glycol hydrophilic coatings reduce bacterial adhesion; silver nanocoatings deliver antibacterial and antioxidant effects but face the problem of leaching and erosion [87]; and AMPS and enzymes, and polyphenols disrupt biofilm signaling pathways and weaken matrix stability, thereby reducing biofilm integrity [88]. This biofilm-mediated antibiotic tolerance arises from three core factors: physical barriers, metabolic adaptation, and changes in the microenvironment. Finally, future biofilm management must integrate traditional methods with advanced molecular technologies, nanotechnology, and biomaterial-based approaches to advance their prevention and control efforts.

The choice of antibiofilm strategies must be closely aligned with clinical contexts, as their effectiveness is influenced by five core factors, including infection type, anatomical location, device involvement, microbial composition, and host immune status. Device-associated infections may benefit from antimicrobial coatings and localized drug delivery systems, whereas chronic wound biofilms may require combined debridement, immune modulation, and matrix-disrupting therapies [89]. Similarly, respiratory biofilm infections often require prolonged combination therapy targeting both microbial persistence and inflammatory dysregulation [90]. Therefore, future clinical management may increasingly rely on personalized and context-specific antibiofilm strategies rather than universal therapeutic approaches.

Biofilms continue to represent one of the most complex challenges in clinical and environmental microbiology due to their complex structure, capacity to evade host defenses, and antimicrobial tolerance. The currently available antibiofilm approaches can be broadly classified as advanced strategies for biofilm eradication and next-generation antibiofilm strategies.

### Advanced strategies for biofilm eradication

A wide range of natural and synthetic molecules, including peptides, enzymes, chelating agents, polyphenols, and small chemical derivatives, can mediate antibiofilm activity. Antibiofilm interventions target all stages of the biofilm life cycle and employ a variety of mechanisms to overcome biofilm-associated tolerance and persistence. These strategies include adhesion inhibition, EPS matrix disruption, quorum-sensing interference, biofilm dispersal induction, combination antimicrobial therapy, nanotechnology-based approaches, phage therapy, and physical or mechanical eradication methods [91].

#### Prevention of adhesion

Surface modification of implants or anti-adhesion coatings effectively inhibits bacterial adhesion and biofilm formation. Andreasson et al. conducted a prospective single-center randomized pilot clinical study to evaluate the clinical safety of Foley catheters fitted with the LubriShield™ coating; it enrolled 30 patients who underwent transurethral resection of bladder tumors, who were divided into a coating group of 14 patients and a standard (non-coating) group of 16 patients. Through follow-up assessments, including urine culture analysis and adverse event monitoring, the study characterized the coating as a permanently bonded ultra-hydrophilic hydrogel carrying antifouling ligands, which releases no antimicrobial agents and exhibits non-antibiotic antibiofilm activity. Study results showed that the two groups had comparable tolerability, with no adverse events reported in the coating group. However, the study had multiple limitations, including a small sample size, a catheter indwelling duration of less than 24 h, a lack of long-term efficacy evaluation, and the absence of significant differences in urinary cultures [92].

#### EPS matrix disruption

Biofilm dispersal represents a promising therapeutic strategy for managing biofilm-associated infections. Many bacteria naturally produce enzymes such as proteases and DNases that mediate biofilm dispersal. This process can also be induced using exogenous agents, including dispersin B, DNase I, proteinase K, and trypsin. Dispersal increases bacterial susceptibility to antibiotics and host immune responses [93]. A preclinical *in vitro* study evaluated the synergistic effects of dispersin B and benzoyl peroxide (BP) against *Cutibacterium acnes/Staphylococcus epidermidis* dual-species biofilms using crystal violet staining, CFU counting, sonication, and sequential treatment assays. Dispersin B degraded poly-N-acetylglucosamine (PNAG), an exopolysaccharide that facilitates both biofilm stability and biocide resistance, disrupting biofilm structure and enhancing BP penetration, while BP exerted oxidative antimicrobial activity. Sequential treatment achieved a >6-log reduction in *C. acnes* viability. Although this study was only designed as an *in vitro* experiment lacking host-related factors, it still supports the potential of dispersin B as an adjuvant anti-biofilm therapeutic option for acne [94].

Other biochemical interventions directly modulate EPS production, including polysaccharides that antagonize biofilm formation in preclinical *in vitro* models. For example, EPS-273 inhibits *P. aeruginosa* biofilm formation and also exhibits antioxidant properties. This study investigated the antibiofilm and anti-infection properties of the marine exopolysaccharide EPS-273 isolated from *Pseudomonas stutzeri* 273 against *P. aeruginosa* PAO1. The compound inhibited biofilm formation and dispersed mature biofilms without affecting bacterial growth. Mechanistically, EPS-273 reduced pyocyanin, exoprotease, rhamnolipid, hydrogen peroxide, and extracellular DNA production, thereby disrupting biofilm stability and virulence. The study concluded that EPS-273 is a promising non-toxic antibiofilm agent possessing strong anti-virulence activity and low cytotoxicity. However, the study was conducted in experimental *in vitro* models, and the long-term stability of this compound has not yet been investigated [95], 96].

EPS-targeting approaches weaken the protective matrix that holds biofilms together. This helps antibiotics penetrate the biofilm and kill the bacteria embedded in it. However, the nature of the biofilm matrix varies among bacterial species, infection sites, and stages of biofilm maturation. Enzymes may be most effective when their specific target is present in large amounts, but less effective against mixed-species biofilms containing different matrix materials. Matrix disruption may also release viable bacteria capable of causing disseminated infections. Therefore, it should be combined with effective antimicrobial treatment. Although *in vitro* results are promising, further animal and clinical studies are needed to address treatment stability, delivery, safety, and regulatory requirements. Therefore, EPS-targeting therapies are more likely to be used as an adjunctive strategy combined with antimicrobial treatments than as stand-alone therapies.

#### Quorum-sensing inhibition

Quorum sensing (QS) is an intercellular communication system that enables bacteria to coordinate biofilm formation and virulence expression. QS inhibition (also called quorum quenching) aims to disrupt biofilm formation and maturation. This approach interrupts bacterial cell-to-cell communication, thereby reducing EPS production, lowering adhesion strength, and enhancing susceptibility to antimicrobials by interfering with signaling molecules such as autoinducers [21], [97], [98], [99]. The researchers have explored such small-molecule inhibitors of quorum sensing, enzymatic degraders of signaling molecules, and biomaterials that release QS inhibitors in a controlled manner. In Gram-negative bacteria, N-acyl-homoserine lactones (AHLs) serve as autoinducers (AIs). The synthetic AHL analogs or structural modifications, such as cyclopentyl or cyclohexanone substitutions, competitively inhibit signaling pathways, thereby attenuating biofilm development in organisms like *Serratia marcescens* and *P. aeruginosa* [100]. Similarly, triazole dihydrofuranone derivatives show both antibiofilm and dispersal properties. Hong et al. conducted a preclinical *in vitro* study on a novel triazole-Schiff base derivative E10, using *S. aureus* and *E. coli* as test targets. Antimicrobial efficacy was evaluated using minimum inhibitory concentration (MIC) assays, time-kill kinetics, crystal violet biofilm inhibition, eradication assays, membrane depolarization and permeability studies, and DNA/protein leakage analyses. E10 derivatives could effectively inhibit biofilm formation in all tested strains and feature low cytotoxicity. However, it has the limitation of incomplete biofilm eradication, and therefore, additional *in vivo* validation data must be supplemented before clinical translation can be achieved [101]. This type of strategy, centered on quorum quenching, has demonstrated great potential for development into a new class of anti-biofilm agents [97]. Currently, two representative methods have emerged in the field: wet-lab experimentation and dry-lab computational research. The first is the preclinical study of hamamelitannin (HAM) by Brackman et al., targeting the TraP QS receptor of *S. aureus*; the team used a combined *in vitro* and *in vivo* model and found that HAM can boost antibiotic efficacy by blocking QS pathways. However, its effects are strain-dependent, with limited activity against *non-S. aureus* strains, so additional clinical validation is still required [98]. The second is the computational pharmacology study of marine-derived halogenated furanones led by Boakye et al., targeting three virulence receptors *LasR, RhlR,* and *PqsR* of *P. aeruginosa*; they applied four computational methods, including molecular docking, molecular dynamics simulations, binding free-energy calculations, and in silico ADME analysis, and found that these furanones have far stronger binding affinity to the first two receptors than to *PqsR*. This approach was rapid and low-cost, but it relies on computational assumptions, possible inaccuracies in predicted interactions, and lacks support from *in vivo* experiments as well. These two studies, one wet-lab and the other dry-lab, complement each other and fully support the application prospects of the quorum-quenching strategy [102]**.**

In comparison with direct bactericidal approaches, QS inhibition offers a potentially attractive anti-virulence strategy that reduces bacterial communication and thereby decreases biofilm formation. Since the QS inhibitors do not directly kill bacteria, they may create less pressure for the development of resistance. QS systems differ among bacterial species and strains, and several communication pathways may operate simultaneously, especially in infections involving multiple species. Therefore, promising results from simple laboratory studies may not translate to a fully established antibiofilm agent. QS inhibitors may be useful for preventing biofilm formation in combination with antibiotics. Further studies in suitable host models, detailed pharmacological evaluation, and clinical trials remain necessary before routine therapeutic application.

#### Inhibition of intracellular signaling pathways

c-di-GMP is a universal bacterial second messenger that regulates the transition between bacteria’s planktonic and biofilm lifestyles [103]. Elevated c-di-GMP promotes biofilm formation, while its degradation by phosphodiesterases facilitates dispersal. Therefore, compounds that reduce intracellular c-di-GMP are being investigated as antibiofilm therapeutics. Small molecules that inhibit diguanylate cyclase (DGC), such as LP-1062, LP-3145, and LP-4010, have shown efficacy against biofilm-forming pathogens, including *P. aeruginosa* and *Acinetobacter baumannii* [104]. This study developed novel genetic tools to investigate c-di-GMP-dependent signaling and biofilm regulation in *P*. *aeruginosa*. For this study, the researchers used fluorescent reporter systems, promoter fusion assays, plasmid-based genetic constructs, intracellular c-di-GMP quantification, and biofilm phenotypic analysis to monitor signaling dynamics *in vivo*. The study focused on c-di-GMP, a bacterial second messenger regulating motility, extracellular polymeric substance production, adhesion, and biofilm maturation. The study concluded that the developed genetic tools effectively enabled real-time monitoring of c-di-GMP signaling and biofilm-associated phenotypes. The study helped to explore *in vivo* (preclinical) analysis and dynamic monitoring of signaling pathways, while this study was constrained by strain-specific differences, technical complexity, and indirect measurement of signaling responses that may not fully represent clinical biofilm conditions [105].

#### Combination antimicrobial therapy

Combination antimicrobial therapy can enhance biofilm elimination through complementary mechanisms of action. Strategies such as antibiotic–enzyme combinations, antibiotic-loaded nanoparticles, and synergistic multidrug regimens improve antimicrobial penetration, disrupt EPS integrity, reduce resistance development, and enhance therapeutic efficacy against persistent biofilm-associated infections. Fowler et al. conducted a phase 3 randomized, placebo-controlled clinical trial (DISRUPT) to evaluate the efficacy and safety of exebacase, a recombinant antistaphylococcal lysin, combined with standard-of-care antibiotics in patients with *S*. *aureus* bacteremia and right-sided infective endocarditis. For the study, randomized patient allocation, intravenous exebacase administration, microbiological confirmation, clinical response assessment, survival analysis, adverse-event monitoring, pharmacokinetic evaluation, and immunogenicity testing were performed. Exebacase lyses the bacterial cell wall, shows synergistic activity with antibiotics, and reduces the development of resistance. This study followed a robust randomized clinical design, comprehensive safety monitoring, and evaluation of combination therapy in clinically relevant infections. However, a highly heterogeneous patient population and a small sample size of the MRSA subgroup were limitations of this study. It ultimately concluded that the combination therapy did not significantly improve patients’ 14-day clinical response, highlighting the design challenges of superiority trials for severe *S. aureus* bloodstream infections. [106]. While natural polyphenols also impair surface integrity and prevent biofilm anchoring. Mazzantini et al. evaluated the anti-staphylococcal antibiofilm activity of a liposomal citrus polyphenol-levofloxacin formulation against *S. aureus* and *S. epidermidis* ocular isolates. For this study, broth microdilution, crystal violet assays, CFU counts, CLSM, and synergistic testing with levofloxacin were employed. This formulation inhibited biofilm formation and reduced the biomass of mature biofilms by affecting EPS production and biofilm maturation (rather than bacterial viability). In this study, quantitative biomass assessment, visualization of biofilm structure, and evaluation of combination therapies were effectively assessed using *in vitro* models. However, the lack of *in vivo* validation and insufficient evidence of antibiotic synergy were the limitations of the study [107].

#### Nanotechnology-based therapies

Nanoparticles (NPs) with customized size, charge, and surface properties can be used to construct a multifunctional anti-biofilm platform. NPs exhibit high surface area-to-volume ratios, enabling efficient drug delivery and enhanced antimicrobial interactions. These platforms commonly use liposomes, metallic nanoparticles, and stimuli-responsive drug delivery to disrupt the biofilms. Their mechanisms include ROS generation, membrane disruption, DNA damage, and metabolic interference [108], 109]. Uusitalo et al. investigated surface-immobilized gold nanorods (AuNRs) as photothermal antibacterial biomaterials against *S. aureus* and *E. coli*. AuNRs were synthesized using a seed-mediated method and immobilized on glass and titanium surfaces through electrostatic interactions. TEM, SEM, XPS, Vis-NIR spectroscopy, thermal imaging, and *in vitro* antibacterial assays under 808 nm near-infrared (NIR) irradiation were used for characterization. The antibacterial mechanism relied on plasmonic heating produced by AuNRs, which disrupted membranes, inhibited the formation of biofilms, and caused localized thermal damage. The glass-supported AuNRs showed significant antibacterial efficacy, whereas titanium mainly absorbed NIR directly. This method showed broad-spectrum, localized antibacterial activity with minimal surface modification. However, dependence on high laser intensity, localized action, potential thermal effects on tissues, and limited *in vivo* validation were the disadvantages of this method. The study concluded that AuNR-based photothermal therapy is a promising strategy for combating biomaterial-associated infections [110]. These innovations demonstrate the shift from passive surfaces to interactive therapeutic systems that are capable of recognizing and responding to infection signals.

The silver nanoparticles (AgNPs) can penetrate microbial membranes to induce cell lysis, and thus exert anti-biofilm activity [111]. The efficacy of nanosilver fluoride (NSF), a composite composed of AgNPs, chitosan, and fluoride, against *S. mutans* biofilms on children’s dental enamel was evaluated in a randomized, double-blind, crossover pilot clinical trial by Freire et al. The trial confirmed that NSF exerts bactericidal effects by producing reactive oxygen species (ROS). This study used various techniques, including TEM, CFU counting, and the simplified oral hygiene index to monitor the changes. The results showed that both *S. mutans* load and the enamel demineralization rate in the NSF group were significantly lower than those in the control group. The study also disclosed four limitations: a small sample size of 32 participants, a follow-up period of 12 weeks, testing only a single caries-causing bacterial species, and no assessment of the risk of long-term silver accumulation. The research ultimately concluded that NSF can act as an effective topical agent for preventing caries in children [112]. Besides NSF, other similar technologies, including polymeric NPs, metal-polymer composites, and ROS-releasing nanomaterials, also hold application prospects [113]. These technologies cover two major sets of application scenarios: preventive uses, such as anti-adhesion coatings and mechanical bactericidal surfaces, and eradication uses, including matrix-penetrating nanoparticles and stimuli-responsive release formulations. Modification strategies that depend on surface engineering to adjust the surface morphology and chemical properties of implants to delay biofilm colonization are also under development. Nanostructured surfaces fabricated via photolithography, when paired with coatings, antibiotics, or nanoparticles, have been shown to deliver significant anti-biofilm efficacy. Currently, the entire field still faces common challenges related to material biocompatibility, cost, durability, and clinical translation and implementation [114], 115]. AgNPs carry long-term application risks, including disruption of the normal oral microbiota, hepatorenal accumulation toxicity, and environmental residue.

#### Phage therapy

Biological and phage-based therapies could serve as alternatives to chemical antibacterial agents. Lytic phages and the depolymerases and lysins they secrete have three core advantages: strain-specific targeted killing, ability to degrade pathogenic bacterial biofilms, and ability to produce synergistic effects with conventional antibiotics. Phages that are genetically modified to express depolymerase activity further improve antibacterial efficacy. However, this type of therapy still faces major challenges such as narrow host coverage, easy induction of phage resistance in pathogenic bacteria, unclear regulatory compliance pathways, and limited *in vivo* delivery efficiency [116], 117]. To overcome these challenges, Karn et al. carried out a randomized, double-blind, placebo-controlled clinical trial. The trial enrolled 60 patients with chronic wounds complicated by MDR biofilm infections, who were evenly divided into two groups of 30 (a treatment group and a placebo group). The Bates-Jensen Wound Assessment Tool was used to evaluate treatment efficacy. The results showed that the treatment group had significantly better sterilization and wound healing outcomes. The targeted therapy precisely combats MDR biofilms, with minimal disruption to normal microbiota, and represents a promising clinical adjuvant regimen. Nevertheless, the study possesses limitations such as small sample size, limited phage screening, high wound heterogeneity, use of concurrent antibiotics, and insufficient long-term follow-ups [118].

Paskhalova et al. conducted a prospective randomized clinical trial, which enrolled 80 adult patients with purulent wounds that varied in etiology and lesion site. The patients were divided into four groups of 20 cases each, and each group received one of four distinct intervention regimens, which included interventions using phages, antibiotics, and negative pressure therapy. They used microbiological, cytological, morphological, electron microscopic, and clinical analyses at multiple postoperative time points. The bacteriophages exerted antibacterial activity through bacteriolytic and bacteriostatic effects, disrupting biofilms, reducing microbial burden, and preventing recolonization. The combined phage instillation with negative pressure therapy showed superior clinical and microbiological outcomes. The method showed advantages like efficacy against MDR infections, enhanced wound regeneration, and advanced safety. However, the study also possesses limitations like heterogeneous wound etiologies, lack of long-term follow-up, and dependence on prior surgical debridement. The study concluded that adjunctive bacteriophage therapy, particularly combined with negative pressure wound therapy, is a promising strategy for managing biofilm-associated purulent wounds [119].

Another class of antibacterial methods with strong application potential is endolysins, which are phage-encoded peptidoglycan hydrolases that can induce bacterial lysis through species-specific action. For instance, Souche et al. evaluated the *in vitro* (preclinical) antibiofilm activity of exebacase (CF-301) against biofilms formed by nineteen *S. epidermidis* clinical isolates from PJIs. For the study, biofilm biomass reduction and viable bacterial counts were assessed after 24 h of exposure and compared with rifampicin, vancomycin, and daptomycin. The exebacase demonstrated significant anti-biofilm and bactericidal activity, with greater efficacy at higher concentrations, and showed synergistic effects when combined with conventional antibiotics. This study evaluated quantitative biomass reduction, bacterial viability, and the effect of combination therapy. However, the study has three major limitations: the observed antibacterial activity varied in an isolate-dependent manner, the research relied mainly on *in vitro* conditions, and the lack of *in vivo* validation of therapeutic efficacy [120].

#### Physical and mechanical approaches

The physical and mechanical approaches disrupt established biofilms through direct removal or destruction. These methods mainly include photodynamic therapy (PDT), debridement, ultrasound-assisted therapy, and cold plasma to reduce biofilm burden, enhance antimicrobial penetration, and improve infection control. PDT exerts a strong bactericidal effect using visible light to activate photosensitizers to generate ROS [121]. Ronqui et al. conducted a preclinical *in vitro* study on methylene blue-mediated PDT, and they found that this approach effectively destroys the structure of mature *S. aureus and E. coli* biofilms. This study used different photosensitizer concentrations and light doses, and treatment efficacy was measured via bacterial colony counting. The study found that the bactericidal effect of PDT exhibited a clear dose-dependent pattern; biofilm strains showed inherent tolerance to PDT when used as a single agent, whereas combining PDT with ciprofloxacin achieved a significant synergistic effect in bactericidal activity. This combined approach improves biofilm penetration and thereby reduces antibiotic tolerance; however, dependence on light penetration, photosensitizer stability, oxygen supply, and the potential complexity of cost associated with combined therapeutic protocols limits its widespread use. [122]. Similarly, Alsaif et al. conducted a randomized controlled *in vivo* clinical study that targeted dental plaque biofilms formed on enamel blocks placed in the oral cavities of 18 healthy adult study participants. They tested the effects of different incubation and irradiation durations for erythrosine-mediated PDT, and found that continuous light irradiation applied after 2-min and 15-min incubation periods achieved a maximum bacterial clearance rate of 95 %. By contrast, the effect of short incubation paired with fractionated light irradiation was far lower. This regimen is non-invasive and has a low incidence of local adverse reactions, but the study only implemented short-term follow-up, so its long-term efficacy remains to be validated [123].

### Next-generation antibiofilm strategies

Next-generation antibiofilm strategies target biofilm-specific mechanisms, such as EPS integrity, quorum sensing, persister cells, and microbial adhesion, using AMPs, smart biomaterials, and host-directed and immune-interactive approaches.

#### Antimicrobial peptides

Some antibacterial agents penetrate the bacterial cytoplasm and interfere with core cellular processes such as DNA replication and protein synthesis. Proline-rich AMPs, including apidaecin and pyrrhocoricin, exert this interference by blocking the activity of ribosome-associated molecular chaperones, inhibiting bacterial proliferation and thereby preventing the expansion of bacterial populations required for biofilm maturation. De Los Santos et al. investigated the activity of Bac7(1–35), a polyproline peptide, against biofilms formed by highly virulent *Klebsiella pneumoniae* using a murine skin abscess *in vivo* (preclinical) model. This study employed CLSM, extracellular polysaccharide aggregation assays, circular dichroism spectroscopy, and UV photodissociation native mass spectrometry to investigate the Bac7(1–35) AMPs. This polyproline peptide induces exopolysaccharide aggregation, kills released dispersed bacterial cells, and triggers the breakdown of biofilm matrices. This study used integrated multi-dimensional analytical techniques to provide detailed mechanistic insights, though their use requires expensive equipment [124].

One of the main strategies for next-generation anti-biofilm approaches is targeting bacterial cell membranes. The AMPs kill bacteria by generating transient pores and modifying lipid arrangements in the cell membrane. Grassi’s et al. used a combination of statistical approaches and machine learning methods to optimize AMP analogs of Temporin 1Tb in a preclinical *in vitro* study. This helped in rapid optimization of peptide structure to increase anti-biofilm activity and screening of synergistic AMPs-EDTA combination. However, this method possesses few limitations, including the higher toxicity of some analogs, lower efficacy against mature biofilms in the absence of an adjuvant, and a lack of *in vivo* clinical validation [125].

#### Host-directed and immune-interactive strategies

Host-directed and immune-interactive strategies are the key pathways for biofilm control. These strategies are beyond classical direct bacterial killing and instead rely on the host pathways to maintain the sterility of medical devices. The researchers have developed an immunomodulatory coating, which recruits macrophages to high-risk, infection-prone sites to enhance phagocytic clearance of bacteria [12]. This technology integrates immune-responsive materials and represents a breakthrough in translational medicine, since sustained drug release is not required. Monoclonal antibodies targeting key polysaccharides significantly reduce biofilm formation by blocking bacterial adhesion and colonization [1]. Burke et al. evaluated the antibiofilm efficacy of the monoclonal antibody TRL1068 against *S. aureus* orthopedic implant infections using a murine spinal implant model (preclinical stage), confocal microscopy, scanning electron microscopy (SEM), fluorescent antibody localization, CFU enumeration, and combination therapy with vancomycin. TRL1068 targets DNABII proteins that stabilize extracellular DNA within the biofilm matrix, thereby disrupting three-dimensional biofilm architecture and releasing sessile bacteria into an antibiotic-susceptible planktonic state. The combined TRL1068–vancomycin therapy significantly reduced implant-associated bacterial burden and restored antibiotic susceptibility. The advantages of the methods include *in vivo* mechanistic visualization, quantitative bacterial assessment, and clinically relevant implant infection modeling. The use of species-specific animal models, the absence of long-term clinical validation, and dependence on adjunctive antibiotic therapy were the limitations of this study [126].

Vaccination represents another promising immunotherapeutic strategy for combating biofilm-associated infections. Vasileiou et al. evaluated a biofilm-embedded bacteria-based vaccine against *Staphylococcus chromogenes*-associated mastitis in sheep. The vaccine contained *S. aureus* bacterin expressing PNAG, a biofilm-associated exopolysaccharide. The vaccine induced significant anti-PNAG antibody production, enhancing opsonization, bacterial clearance, and inhibition of biofilm persistence and adhesion. The vaccinated sheep showed reduced bacterial load, milder inflammation, and shorter disease duration. This method showed comprehensive *in vivo* immune-response evaluation and biofilm-targeted immunotherapy assessment. However, limited strain specificity and the need for larger-scale clinical validation were the limitations of the study [127].

#### Smart and responsive systems-based antibiofilm agents

The development of smart and responsive systems represents an emerging trend in next-generation antibiofilm strategies, offering targeted and stimuli-responsive therapeutic interventions. These devices employ smart materials that respond to biological signals. Specifically, the stimuli-responsive coatings release antibiofilm agents upon sensing environmental changes such as a pH drop, enzyme secretion, or oxidative stress. This controlled release ensures targeted therapy and minimizes systemic toxicity. Researchers have also developed a pH-sensitive hydrogel coating that releases antibiotics in response to acidification caused by infection [128]**.** Gherasim et al. conducted an *in-vitro* experimental study (preclinical) to investigate MAPLE (Matrix-Assisted Pulsed Laser Evaporation) coatings embedded with magnetite nanoparticles conjugated with essential oil for antibiofilm applications on biomedical surfaces. This study employs magnetite nanoparticles as an essential oil carrier to achieve controlled release of antibacterial components. Essential oils exert antibacterial effects by disrupting microbial cell membranes, interfering with quorum sensing, and inhibiting bacterial adhesion and biofilm maturation.

The MAPLE technique enables uniform coating deposition while preserving the stability of bioactive compounds, thereby ensuring sustained release of antibacterial agents and reduced microbial colonization. But there are still major challenges in this field, such as lack of *in vivo* validation, insufficient formulation stability, high preparation complexity, and poor long-term durability [128]. In our opinion, future research needs to shift from passive antibacterial surfaces to smart responsive systems that can detect infection-related biochemical changes and autonomously release targeted therapeutic agents. The real-world implementation of this approach requires integration of three core technologies: biosensing, nanotechnology, and artificial intelligence.

#### Clustered regularly interspaced short palindromic repeats (CRISPR) based genome editing approaches as antibiofilm strategies

CRISPR-based antibacterial biofilm approaches focus on precisely targeting and modifying bacterial genes involved in biofilm formation, virulence, quorum sensing, and antibiotic resistance. Kang et al. used CRISPR-Cas9 genome editing (an *in vitro* preclinical study) to investigate the role of the quorum-sensing gene *luxS* in biofilm formation by the clinical isolate *E. coli* SE15, a major pathogen causing catheter-associated urinary tract infections. The research team used CRISPR-Cas9 to eliminate the quorum-sensing gene luxS, achieving an editing efficiency of 22.7 %. Biofilm assays and quantitative real-time PCR confirmed that the *luxS* mutant strain saw significant reductions in both its biofilm formation level and the expression of three categories of biofilm-related genes, namely *mqsR, pgaBC*, and *csgEF*. The study demonstrates that this approach has precise targeting but suffers from low editing efficiency. It ultimately concluded that CRISPR-Cas9 is a highly promising tool for developing targeted anti-biofilm therapies [129].

#### Antibiofilm strategies based on artificial intelligence and machine-learning models

In recent years, multiple studies have highlighted that artificial intelligence and machine learning models have played an increasingly important role in biofilm diagnosis, feature analysis, and treatment optimization. Various mainstream technologies, including convolutional neural networks (CNNs), recurrent neural networks (RNNs), long short-term memory (LSTM) models, support vector machines (SVMs), transformer architectures, and U-Net/ResNet image-analysis systems, interface with multi-source datasets such as microscopic imaging, genomic, and proteomic datasets to complete the detection and classification of biofilms. These technologies help to perform high-throughput screening of anti-biofilm compounds. While these methods offer advantages such as rapid, automated, and sensitive analysis with predictive capabilities, they still have limitations, including requirements for high-quality data and computational resources, as well as insufficient interpretability and generalizability. Overall, these studies demonstrate that AI-driven biofilm diagnostics and therapeutic modeling represent promising tools for the precision management of biofilm-associated infections [130], 131] (Table 3). Table 3 shows the translational framework for major biofilm-targeted therapies

**Table 3:** Translational framework for major biofilm-targeted therapies.

Strategy	Primary mechanism of action	Target biofilm stage	Mono-/multispecies suitability	Clinical application	Evidence level	Primary advantage	Major translational barrier	Clinical challenge	Regulatory/manufacturing issue	Key missing evidence
Antimicrobial coatings [92]	Prevention of microbial adhesion and colonization	Surface adhesion	Both	Catheters, orthopedic implants, medical devices	Clinical	Prevents biofilm establishment before infection	Durability and long-term efficacy	Device colonization under prolonged use	Sterilization compatibility and coating stability	Long-term real-world performance studies
Enzymatic dispersal agents [93], [94], [95], [96]	EPS matrix degradation and biofilm dispersal	Mature biofilms	Both	Wounds, implants, device-associated infections	Preclinical	Disrupts EPS matrix and improves antimicrobial access	Stability and delivery	Effective dispersal of mature biofilms	Product formulation and storage stability	Clinical validation studies
Quorum-quenching/quorum-sensing inhibitors [21], [97], [98], [99], [100], [101], [102]	Disruption of microbial communication	Maturation	Species or class-specific	Chronic biofilm-associated infections	Preclinical	Reduces virulence	Limited clinical validation	Variable efficacy among pathogens	Regulatory approval pathway	Clinical efficacy and safety data
Intracellular signaling inhibitors [103], [104], [105]	Disruption of cyclic-di-GMP and other biofilm-regulatory signaling networks	Biofilm formation and maturation	Both	Chronic biofilm-associated infections	Preclinical	Reduces biofilm formation and promotes dispersal without direct bactericidal pressure	Limited target specificity and *in vivo* validation	Variable signaling pathways among pathogens	Drug optimization and regulatory approval	Clinical efficacy and long-term safety studies
Combination therapies [106], 107]	Simultaneous targeting of multiple biofilm mechanisms	Multiple stages	Both	Chronic polymicrobial infections	Clinical/preclinical	Simultaneously targets multiple biofilm mechanisms	Optimization and standardization	Treatment complexity	Protocol standardization	Comparative effectiveness studies
Implant nano-topographies [108], [109], [110]	Surface-mediated inhibition of bacterial attachment	Surface adhesion	Both	Orthopedic and dental implants	Preclinical/early clinical	Antibiotic-free inhibition of bacterial adhesion	Mechanical stability	Implant integration and tissue compatibility	Manufacturing reproducibility	Long-term clinical validation
Nanoparticles [111], [112], [113], [114], [115]	Enhanced drug delivery and matrix penetration	Mature biofilms	Both	Device infections, chronic wounds	Preclinical/early clinical	Enhanced drug delivery and improved biofilm penetration	Toxicity and biodistribution	Effective penetration into dense biofilms	Scale-up production and batch consistency	Long-term safety and efficacy data
Phage therapy [116], [117], [118], [119], [120]	Bacterial lysis and EPS degradation	Mature biofilms	Species-specific	Multidrug-resistant and chronic infections	Clinical/multi-center trial stage	High specificity against target pathogens	Narrow host range and immune clearance	Polymicrobial infections	Regulatory classification and standardization	Standardized randomized clinical trials
Photodynamic therapy (PDT) [121], [122], [123]	ROS-mediated oxidative killing	Mature biofilms	Both	Oral biofilms, chronic wounds, localized infections	Preclinical/early clinical	Localized antimicrobial activity with low resistance potential	Limited light penetration	Deep tissue infections	Treatment standardization	Large multicenter clinical trials
Antimicrobial peptides (AMPs) [124], 125]	Membrane disruption and antimicrobial activity	Mature biofilms	Both	Wounds and topical infections	Preclinical	Broad-spectrum and rapid antimicrobial activity	Stability and host toxicity	Rapid degradation *in vivo*	Manufacturing cost and formulation challenges	Human efficacy and safety studies
Host-directed therapies [1], 126], 127]	Immune modulation and enhancement of host defense	Multiple stages	Both	Chronic and recurrent infections	Preclinical	Enhances host immunity and infection clearance	Safety and immune variability	Patient heterogeneity	Long-term safety assessment	Human translational studies
Smart/responsive systems [128]	Triggered drug release and adaptive therapy	Multiple stages	Both	Device-associated infections	Preclinical	Controlled and site-specific therapeutic release	Manufacturing complexity	Reliable activation *in vivo*	Scalability and quality control	Clinical feasibility studies
CRISPR-based approaches [129]	Targeted disruption of biofilm-associated genes and antimicrobial resistance determinants	Multiple stages	Species-specific	Multidrug-resistant and recurrent biofilm infections	Preclinical	Highly precise and programmable targeting of pathogens and resistance genes	Efficient delivery to biofilms and off-target effects	Delivery across dense biofilm matrices	Manufacturing complexity and regulatory uncertainty for gene-editing therapeutics	*In vivo* validation, safety assessment, and clinical translation studies
Antibiofilm strategies based on artificial intelligence and machine-learning models [130], 131]	Predictive modeling and therapeutic optimization	Not available	Both	Precision antibiofilm therapy, drug discovery, device-associated infections, and clinical decision support	Preclinical/early clinical	Accelerates drug discovery and precision therapy	Limited high-quality training datasets	Clinical integration and model interpretability	Algorithm validation and regulatory approval	Prospective multicenter clinical studies

### Translational framework for antibiofilm therapy development

The successful translation of biofilm research into clinically effective therapies requires a systematic understanding of biofilm-associated vulnerabilities and their potential as therapeutic targets. Rather than viewing biofilms solely as structural microbial communities, a translational approach should identify actionable biological targets, evaluate available intervention strategies, assess evidence quality, and recognize barriers to clinical implementation [132], 133]. For example, EPS matrices can be targeted using enzymatic dispersal agents, nanoparticles, and matrix-degrading compounds; however, limited *in vivo* validation and regulatory challenges continue to hinder widespread adoption [4].

Although numerous antibiofilm strategies have demonstrated promising activity *in vitro*, their translational maturity varies considerably. Approaches such as antimicrobial coatings, debridement, and antibiotic lock therapy have progressed toward clinical implementation [134]. In contrast, quorum-sensing inhibitors, engineered bacteriophages, CRISPR-based technologies, and nanomaterial-based systems remain largely preclinical [100], 118], 120], 128], 129]. Importantly, evaluation of antibiofilm technologies should extend beyond mechanistic efficacy and include evidence quality, translational feasibility, and infection-specific clinical applicability. In our opinion, a decision-making framework that integrates biofilm characteristics, therapeutic vulnerabilities, levels of evidence, translational barriers, and clinical utility could help in prioritizing and screening clinical strategies with high translational potential.

## Translational challenges of antibiofilm strategies from laboratory research to clinical and device settings

Despite the advances in current antibiofilm research, the successful clinical translation of research outcomes still faces major challenges. This is mainly due to the complex and heterogeneous nature of biofilm-associated infections. The real-world implementation of these emerging anti-biofilm technologies requires meeting prerequisites such as safety, biocompatibility, scalability, regulatory approval, and reliable performance in different clinical settings [135]. Biofilms cause recurrent infections and therapeutic failure in implant-associated infections, chronic non-healing wounds, cystic fibrosis-associated respiratory infections, and catheter-associated bloodstream infections [136], [137], [138]. The key barriers and opportunities for clinical translation of antibiofilm strategies from the laboratory to the clinic are described in the following sections.

### Translational bottlenecks limiting clinical implementation

Despite considerable progress in anti-biofilm technologies, their clinical translation remains limited by several challenges specific to biofilm-related infections. Different therapeutic platforms face distinct translational barriers [68], 139]. This review article identifies exclusive translation barriers for three categories of technology platforms: a) antibacterial coatings and implant nanotopographies must maintain long-term performance requirements under physiological conditions, while also being compatible with manufacturing and sterilization processes [92]; b) nanoparticle systems and photodynamic therapies often encounter delivery difficulties, such as insufficient tissue penetration and toxicity [110], 112], 123]; c) phage-based and enzyme-based dispersal therapies must overcome obstacles including immune responses, narrow activity spectra, formulation stability, and regulatory classification issues [118]. Moreover, the absence of standardized diagnostic tools, lack of biomarkers, and the difficulty of choosing clinically appropriate randomized trials still limit clinical evaluation. These bottlenecks should be resolved for translating laboratory research results into clinically available therapies.

### Bridging laboratory models and clinical reality

The major obstacle in the clinical translation of anti-biofilm therapies is a complexity mismatch between the biofilm models employed in laboratory research and real-world infections. Most emerging anti-biofilm strategies have demonstrated promising therapeutic potential in standard *in vitro* systems but have failed consistently in clinical translation. This is mainly because these models do not consider important aspects of human infections such as host immune responses, polymicrobial infections, physiological heterogeneity, nutrient and oxygen gradients, device-associated microenvironments, and biofilm maturation dynamics [68], 139]. For example, the highly dynamic biofilm environment on the surface of indwelling medical devices is difficult to replicate in laboratory settings; such simplified systems overestimate the actual clinical efficacy of tested therapies. Future progress, therefore, depends on the development and standardization of clinically relevant, host-integrated, and polymicrobial biofilm models that accurately mimic the internal host environment and improve the prediction of therapeutic outcomes.

### Early detection and diagnostic integration

Early recognition of biofilm formation plays a pivotal role in clinical translation. Evidence suggests that traditional diagnostic methods often fail to identify sessile communities of bacteria (those attached to surfaces such as catheters or implants) because they rely on the detection of planktonic bacterial cells. Therefore, clinical microbiology laboratories need to integrate molecular and imaging technologies to detect biofilm-associated infections more effectively. Several studies have demonstrated that imaging-assisted diagnostics, combined with molecular markers, help in the early identification of biofilm colonization before irreversible device contamination occurs [1], 108], 140]. The importance of integrating diagnostics into clinical workflows will help in early interventions before the formation of mature biofilms. The bedside biosensors and fluorescent probes help in early biofilm detection. For instance, a surface plasmon resonance biosensor developed by Bajaj A et al. can deliver accurate real-time detection, assist in determining the optimal timing for replacing medical equipment, and improve patient prognosis. [141].

### Regulatory and safety considerations

To achieve regulatory translation, anti-biofilm medical devices must maintain three core requirements: reproducibility, long-term safety, and stable mass production, and their clinical trials must comply with two standards: antibacterial performance and biocompatibility. The two major current regulatory frameworks, ISO 10993 and the U.S. Food and Drug Administration (FDA) framework, mandate obligatory assessment of surface toxicity, particle leaching, and immune activation potential. Highmore CJ et al. note that regulatory bottlenecks have delayed the market launch of these devices. The Biofilm Research-Industrial Engagement Framework (BRIEF) proposed guarantees global data comparability through inter-laboratory verification [68]. These transparent regulatory guidelines could help to accelerate the translation of promising antibiofilm technologies into approved clinical devices.

### Clinical workflow and device management

Clinical teams should integrate antibiofilm technologies through updated infection-control protocols. They train healthcare staff to detect early indicators of device colonization and recommend adopting evidence-based guidelines for device insertion, maintenance, and removal. For example, hospitals implement catheter-care bundles (a standardized set of clinical practices designed to prevent infections associated with catheter use) that include the use of coated devices, sterile insertion techniques, and scheduled replacement cycles. In orthopedic surgery, surgeons mainly select implants with embedded antibiofilm coatings to minimize postoperative infections [142]. This type of intervention can directly translate laboratory formulations into substantial patient benefits.

### Real-world implementation and monitoring

Continuous monitoring and evaluation of the translated antibiofilm technologies ensure they remain effective under routine use. Hospitals should collect data on infection incidence, device longevity, and adverse reactions to evaluate long-term outcomes. It is worth noting that continuous feedback between clinicians and developers helps to develop next-generation coating formulations. It was observed that the use of silver-coated catheters across multiple intensive-care units resulted in a sustained, significant reduction in infection rates after one year [143]**.** Such real-world evidence validates laboratory innovations and encourages broader clinical adoption.

### Future directions and realistic pathways for clinical translation

The core prerequisite for the successful clinical translation of anti-biofilm technology relies on interdisciplinary collaboration across microbiology, materials science, clinical medicine, and regulatory agencies. Therefore, the researchers must aim to develop standardized biofilm testing methods that simulate clinical scenarios, establish connections between diagnostic data and targeted delivery systems, develop biodegradable coatings with temporary anti-biofilm protective functions, and integrate artificial intelligence and machine learning models to enable infection prediction and device monitoring. The successful management of biofilm-associated infections requires the adoption of an integrated, phased, adaptive therapeutic system to replace the traditional approach that relies on a single antimicrobial agent. The biofilm life cycle exhibits dynamic structural and metabolic heterogeneity consisting of four stages, including adhesion, maturation, persistence, and dispersal phases; each phase is characterized by distinct physiological and molecular features [2], 144]. Consequently, single-target therapies have limited efficacy and are prone to recurrences or incomplete eradication [145]. Future antibiofilm strategies should therefore incorporate multimodal interventions targeting EPS integrity, quorum-sensing pathways, persister-cell survival, microbial adhesion, and host inflammatory responses (Figure 3) [133], 146]. In this context, adaptive systems integrating smart-responsive biomaterials, nanotechnology-assisted delivery platforms, enzymatic dispersal agents, immune-modulatory coatings, and precision-targeted therapeutics are increasingly recognized as promising translational approaches for improving therapeutic efficacy and reducing antimicrobial tolerance in complex biofilm-associated infections [147].

**Figure 3:**
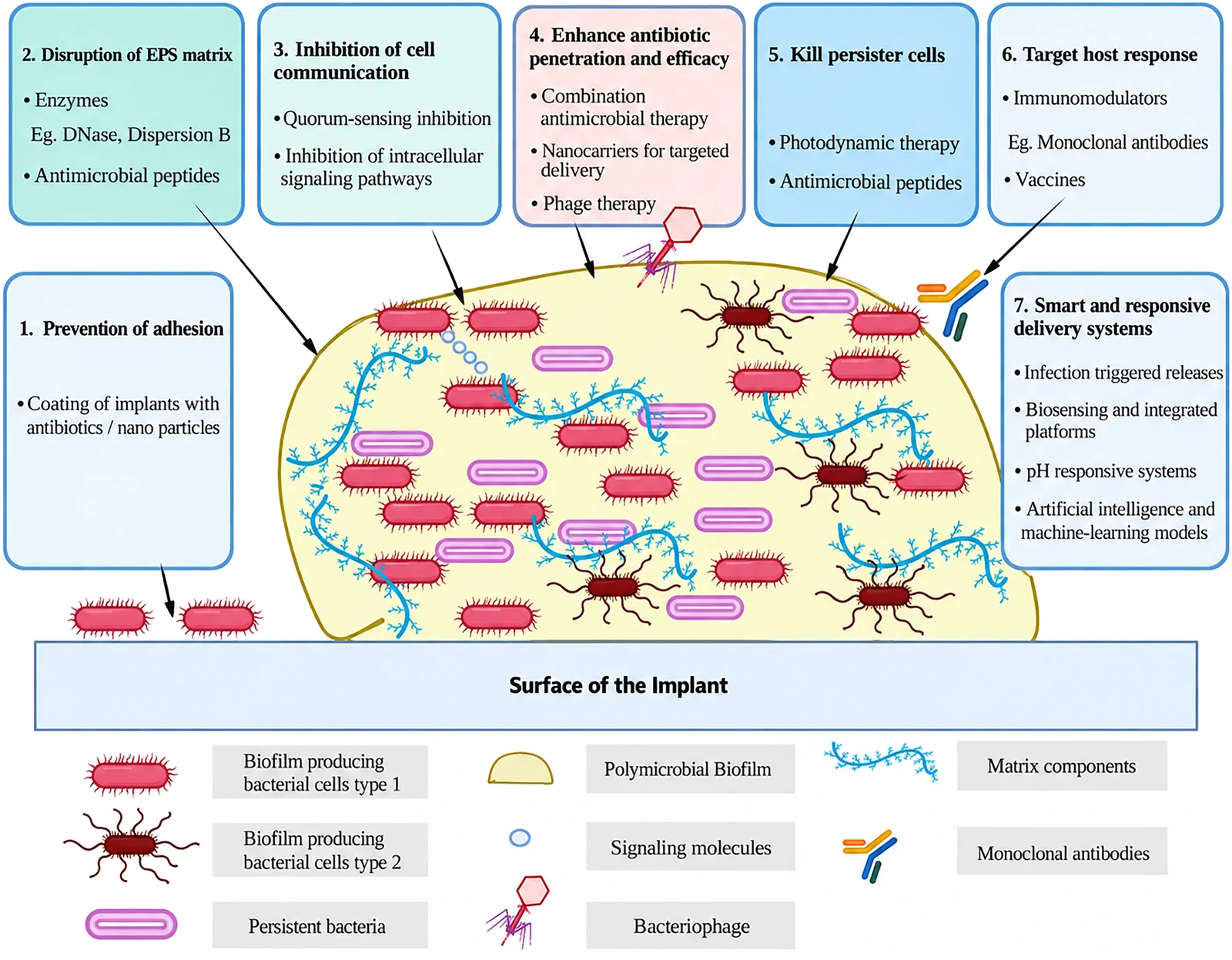
Multimodal and stage-specific strategies targeting key vulnerabilities across the biofilm life cycle. Microbial adhesion to implant surface may be prevented by antimicrobial coatings and surface modifications. Established biofilms may be disrupted using matrix-degrading enzymes, antimicrobial peptides, quorum-sensing inhibitors, and intracellular signaling modulators. Combination therapies, nanocarriers, and bacteriophages enhance antimicrobial delivery, while photodynamic therapy targets persistent cells. Host-directed therapies and smart-responsive delivery systems, including AI-assisted platforms, represent emerging strategies for precision biofilm management. Created in BioRender. Pottathil, S. (2026) https://BioRender.com/ufptuhy (accessed on 9 June 2026).

The successful clinical translation of antibiofilm therapies requires a multidisciplinary pathway that connects experimental discoveries to clinical implementation, which must be established. First, through preliminary mechanistic studies, researchers must identify molecular targets associated with biofilm formation, persistence, quorum sensing, EPS integrity, and antimicrobial tolerance [148]. Next, validate the target in preclinical models that replicate the core characteristics of human infections. Before clinical application, complete the assessments of safety, toxicity, and biocompatibility of the antibiofilm agents. Then, confirm therapeutic efficacy, optimize treatment strategies, and evaluate patient-centered outcomes across diverse infection settings through well-designed clinical trials. Translation further depends on regulatory approval, manufacturing reproducibility, and standardized treatment protocols [146], 149]. Finally, long-term post-marketing surveillance must be implemented to monitor long-term therapeutic performance, antimicrobial resistance trends, device compatibility, and rare adverse events. This phased process represents a feasible framework that bridges basic research and clinical application.

## Conclusions and future perspectives

Bacterial biofilms pose a major healthcare problem due to their high tolerance to antimicrobial agents, complex structure, and ability to colonize medical devices. Current approaches such as surgical debridement, systemic antibiotics, and antimicrobial coatings may be limited in their effectiveness depending on the type and site of infection. Emerging strategies such as quorum-sensing inhibition, photodynamic therapy, combination therapies, and smart-responsive systems have shown promising antibiofilm activity, but most of the evidence is still preclinical. In addition, the commonly used laboratory models do not fully simulate the complexity of biofilm infections in patients. Future research should therefore focus on more clinically relevant polymicrobial and host-integrated models, standardized testing methods, and clinical studies adapted to specific biofilm infections. Combining different therapeutic approaches and targeting different stages of biofilm development may improve biofilm management, but further studies are needed to establish their safety, effectiveness, long-term performance, and clinical feasibility before routine clinical implementation.
